# Chromatin Profiling of the Repetitive and Nonrepetitive Genomes of the Human Fungal Pathogen Candida albicans

**DOI:** 10.1128/mBio.01376-19

**Published:** 2019-07-23

**Authors:** Robert Jordan Price, Esther Weindling, Judith Berman, Alessia Buscaino

**Affiliations:** a Kent Fungal Group, School of Biosciences, University of Kent, Canterbury, Kent, United Kingdom; b Department of Microbiology and Biotechnology, George S. Wise Faculty of Life Sciences, Tel Aviv University, Ramat Aviv, Israel; Universidad de Córdoba

**Keywords:** *Candida albicans*, euchromatin, genome instability, heterochromatin, chromatin, epigenetics

## Abstract

Eukaryotic genomes are packaged into chromatin structures that play pivotal roles in regulating all DNA-associated processes. Histone posttranslational modifications modulate chromatin structure and function, leading to rapid regulation of gene expression and genome stability, key steps in environmental adaptation. Candida albicans, a prevalent fungal pathogen in humans, can rapidly adapt and thrive in diverse host niches. The contribution of chromatin to C. albicans biology is largely unexplored. Here, we generated the first comprehensive chromatin profile of histone modifications (histone H3 trimethylated on lysine 4 [H3K4me^3^], histone H3 acetylated on lysine 9 [H3K9Ac], acetylated lysine 16 on histone H4 [H4K16Ac], and γH2A) across the C. albicans genome and investigated its relationship to gene expression by harnessing genome-wide sequencing approaches. We demonstrated that gene-rich nonrepetitive regions are packaged into canonical euchromatin in association with histone modifications that mirror their transcriptional activity. In contrast, repetitive regions are assembled into distinct chromatin states; subtelomeric regions and the ribosomal DNA (rDNA) locus are assembled into heterochromatin, while major repeat sequences and transposons are packaged in chromatin that bears features of euchromatin and heterochromatin. Genome-wide mapping of γH2A, a marker of genome instability, identified potential recombination-prone genomic loci. Finally, we present the first quantitative chromatin profiling in C. albicans to delineate the role of the chromatin modifiers Sir2 and Set1 in controlling chromatin structure and gene expression. This report presents the first genome-wide chromatin profiling of histone modifications associated with the C. albicans genome. These epigenomic maps provide an invaluable resource to understand the contribution of chromatin to C. albicans biology and identify aspects of C. albicans chromatin organization that differ from that of other yeasts.

## Abstract (precis)

The fungus Candida albicans is an opportunistic pathogen that normally lives on the human body without causing any harm. However, C. albicans is also a dangerous pathogen responsible for millions of infections annually. C. albicans is such a successful pathogen because it can adapt to and thrive in different environments. Chemical modifications of chromatin, the structure that packages DNA into cells, can allow environmental adaptation by regulating gene expression and genome organization. Surprisingly, the contribution of chromatin modification to C. albicans biology is still largely unknown. For the first time, we analyzed C. albicans chromatin modifications on a genome-wide basis. We demonstrate that specific chromatin states are associated with distinct regions of the C. albicans genome and identify the roles of the chromatin modifiers Sir2 and Set1 in shaping C. albicans chromatin and gene expression.

## INTRODUCTION

Packaging of genomes into chromatin is the key determinant of nuclear organization (1, 2). The basic unit of chromatin is the nucleosome, consisting of a histone octamer of two molecules each of histone H2A, H2B, H3, and H4, around which 147 bp of DNA are wrapped in almost two complete turns (3). Histone proteins are subjected to a wide variety of posttranslational modifications, known as histone marks, that decorate distinct chromatin regions (3). Modification of chromatin structure controls a plethora of nuclear processes, including gene expression and DNA repair and replication (3, 4). Consequently, genome-wide maps of histone modifications have been instrumental in identifying functionally different regions of eukaryotic genomes (5, 6).

Gene-rich, nonrepetitive DNA is associated with active histone marks, forming euchromatin, a chromatin state permissive to transcription and recombination (7). At euchromatic regions, promoters of active genes are enriched in histone H3 trimethylated on lysine 4 (H3K4me^3^) and acetylated on lysine 9 (H3K9Ac), while coding sequences are enriched in a different set of histone modifications, such as acetylated lysine 16 on histone H4 (H4K16Ac) (8–10). In contrast, genomic regions enriched in repetitive DNA and low in gene density are assembled into heterochromatin (7).These repetitive sequences (including tandem repeats, transposable elements, and gene families) are a threat to genome stability. At repetitive elements, heterochromatin assembly promotes genome stability by repressing deleterious recombination events (7, 11, 12). Heterochromatin is devoid of active histone marks (i.e., H3K4me^3^, H3K9Ac, and H4K16Ac) and is enriched in repressive histone marks such as methylation of lysine 9 on histone H3 (H3K9me) and methylation of lysine 27 on histone H3 (H3K27me) (1).

While euchromatin structure is largely conserved across organisms, histone marks associated with heterochromatic regions differ between organisms. For example, in the model system Saccharomyces cerevisiae, heterochromatin is devoid of H3K9me and H3K27me marks but contains hypomethylated H3K4 and hypoacetylated H3K9 and H4K16 (1, 13). Phosphorylation of serine 129 on histone H2A (known as γH2A) is enriched in heterochromatin regions in S. cerevisiae, Schizosaccharomyces pombe, and Neurospora crassa, in a cell cycle-independent manner (14–17). Since γH2A is a hallmark of DNA double-strand breaks (DSBs), these findings suggest that heterochromatic regions are flagged for DNA damage. In contrast, in human cells, phosphorylation of H2AX, a modification functionally analogous to the γH2A modification, does not decorate heterochromatic regions (18, 19).

Chromatin modifications also play major roles in controlling genome stability by dictating pathways of DNA repair. Indeed, choices of DNA repair pathways (i.e., nonhomologous end joining [NHEJ] or homologous recombination [HR]) depend on the chromatin state of the genomic region undergoing repair. Furthermore, extensive chromatin changes, including recruitment of γH2A, are linked to repair of DNA breaks (20). Consequently, in unchallenged cells, γH2A mapping is used to identify unstable genomic regions (named γ-sites) that are prone to intrinsic DNA damage and recombination (14).

Chromatin modifications are reversible, and specific histone modifiers maintain or erase the histone modification state associated with different chromatin regions. Among these, histone acetyltransferases (HATs) and histone deacetylases (HDACs) maintain and erase histone acetylation, respectively, while histone methyltransferases (HMTs) and demethylases (HDMs) are responsible for the methylation state of histones (2, 3). These chromatin modifications rapidly and reversibly alter gene expression and genome stability and therefore can have a major impact on the responses of microbial organisms in adapting rapidly to sudden environmental changes (21, 22).

One such microorganism is the human fungal pathogen Candida albicans. C. albicans is a benign commensal organism that colonizes the mouth, the skin, and the urointestinal and reproductive tracts of most individuals. However, C. albicans is also the most common causal agent of invasive fungal infections, and systemic infections are associated with high mortality rates (up to 50%) (23). C. albicans is such a successful opportunistic pathogen because it rapidly adapts to and thrives in diverse host niches. Its ability to switch among multiple specialized cell types and its remarkable genomic plasticity underpin C. albicans adaptation (24).

The C. albicans genome has 8 diploid chromosomes that contain 6,408 annotated protein-coding genes, as well as a large number of noncoding RNAs (ncRNAs) (25–28). The genome also contains several classes of repetitive elements: telomeres/subtelomeres, the ribosomal DNA (rDNA) locus, major repeat sequences (MRSs), and transposable elements (29). Telomeres are composed of tandemly repeating 23-bp units, while subtelomeres are enriched in long terminal repeats (LTR), retrotransposons, and gene families, such as the *TLO* genes (29, 30).

The rDNA locus consists of a tandem array of ∼12-kb units repeated 50 to 200 times. Each unit contains the two highly conserved 35S and 5S rRNA genes that are separated by nontranscribed spacer region 1 (NTS1) and NTS2, whose sequences are not conserved with other eukaryotes (29, 31).

MRS loci are long (10-to-100-kb) tracts of nested DNA repeats found on 7 of the 8 C. albicans chromosomes (29, 32). These repetitive domains, found in C. albicans and in the closely related species C. dubliniensis and C. tropicalis, are formed by large tandem arrays of 2.1-kb repetitive DNA sequence (RPS) units flanked by nonrepetitive HOK and RBP-2 elements. Each RBP-2 element contains a protein-coding gene, *FGR6*, important for morphological switching (32, 33).

Several classes of retrotransposons are present in the C. albicans genome, including 16 classes of LTR retrotransposons (Tca1 to Tca16) and Zorro non-LTR retrotransposons that are present in 5 to 10 copies per cell, dispersed along the chromosomes. Among those, Tca2, Tca4, Tca5, Zorro-2, and Zorro-3 are capable of transposition (34–36).

The C. albicans genome is remarkably plastic, and natural isolates exhibit a broad spectrum of genomic variations, including loss of heterozygosity (LOH) events, chromosome rearrangements, and aneuploidy (37). Evolution experiments and analyses of clinical isolates have demonstrated that repetitive elements represent hypermutable sites of the C. albicans genome and are prone to high rates of recombination (37, 38).

Regulation of chromatin structure plays critical roles in regulating C. albicans gene expression and genome instability (39–42). However, comprehensive profiling of histone modifications across the whole C. albicans genome is still lacking. Generation of these epigenomic maps will be essential to truly understand the impact of chromatin regulation on C. albicans adaptation and on development of virulence traits.

In this study, we used chromatin immunoprecipitation with massively parallel deep sequencing (ChIP-seq) technology to establish the first comprehensive genome-wide map of C. albicans histone modifications (H3K4me^3^, H3K9Ac, H4K16Ac, and γH2A), marking euchromatic and heterochromatic regions and potential recombination-prone unstable sites. Genome-wide mapping of RNA polymerase II (RNAPII) and transcriptome expression profiling unveiled the link between histone modification states and transcriptional activity. We demonstrate that specific chromatin states are associated with the repetitive and nonrepetitive C. albicans genomes. While gene-rich regions are associated with active chromatin marks mirroring their transcriptional state, different types of repetitive elements are assembled into distinct chromatin types. Finally, we present the first C. albicans quantitative ChIP-seq (q-ChIP-seq) methodology, which has permitted us to elucidate the roles of HDAC Sir2 and HMT Set1 in shaping the chromatin state of C. albicans genome and regulating gene expression.

## RESULTS

### Genome-wide histone modification profiling in C. albicans.

The C. albicans genome contains two homologous pairs of divergently transcribed histone H2A and H2B genes as well as histone H3 and H4 genes in addition to a single histone H3 gene (see Fig. S1A in the supplemental material). Sequence alignment demonstrated that the frequently modified amino acid residues H3K4, H3K9, H4K16, and H2AS129 are conserved in C. albicans (Fig. S1B).

10.1128/mBio.01376-19.1 FIG S1 (A) (Top) Diagram of genome organization of C. albicans histone genes. (Bottom) Histone gene names and locations, according to assembly 22. (B) Alignment of C. albicans H2A, H3, and H4 protein sequences against S. cerevisiae and S. pombe homologues. Residues highlighted in red were investigated in this study and are conserved across all species. Download FIG S1, TIF file, 0.9 MB. Copyright © 2019 Price et al. 2019 Price et al. This content is distributed under the terms of the Creative Commons Attribution 4.0 International license.

To explore the chromatin signature of C. albicans repetitive and nonrepetitive regions, we globally mapped the genomic locations of H3K4me^3^, H3K9Ac, H4K16Ac, and γH2A by performing chromatin immunoprecipitation followed by high-throughput sequencing (ChIP-seq). Since nucleosomes are not equally distributed across genomes, we accounted for nucleosome occupancy by performing genome-wide profiling of unmodified histone H3 and histone H4. To correlate specific histone modification profiling with transcriptional activity, we performed genome-wide mapping of RNA polymerase II (RNAPII) occupancy. We also performed transcriptome analysis by strand-specific RNA sequencing (RNA-seq) to profile gene expression levels.

**FIG 1:**
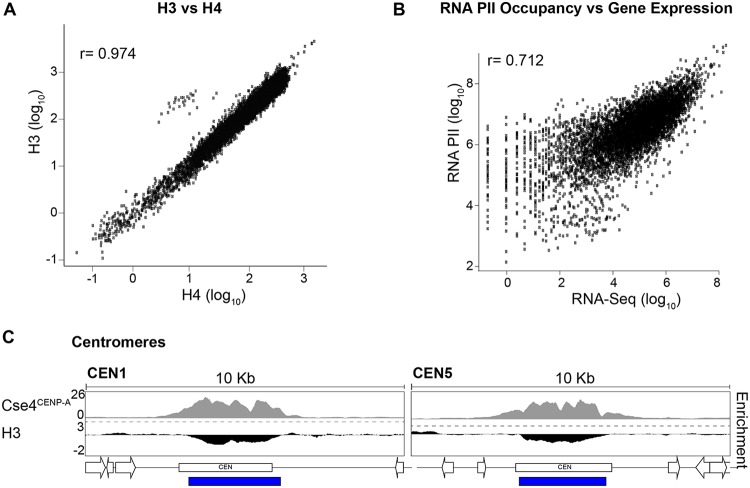
Histones and RNA polymerase II occupancy. (A) Correlation between H3 and H4 occupancy (log_10_ counts at 1-kb bins) across the C. albicans genome. (B) Correlation between RNAPII occupancy (log_10_ counts) and transcriptional levels (RNA-seq; log_10_ counts) at protein-coding genes. (C) Histone H3 is depleted at centromeric regions. Data indicate fold enrichment (log_2_) of histone H3 relative to unmodified H4 across CEN1 (Chr1) and CEN5 (Chr5) centromeric and pericentromeric regions in C. albicans. The Cse4^CENP-A^ enrichment profile (72) is shown for comparison. The blue bar indicates statistically significantly depleted regions for histone H3.

ChIP-seq was performed using C. albicans wild-type (WT) cells grown under standard laboratory growth conditions (YPAD [1% yeast extract, 2% peptone, 2% dextrose, 0.1 mg/ml adenine, 0.08 mg/ml uridine], 30°C) using antibodies specific for modified or unmodified histones. Input (I) samples and immunoprecipitated (Ip) samples were sequenced using an Illumina HiSeq2000 platform (single-end 50-bp reads; average coverage, 28×; see Table S2 in the supplemental material) and aligned to a custom haploid version of assembly 22 of the C. albicans genome (25). Unmodified histone H3 occupancy showed a strong positive correlation with histone H4 occupancy (Pearson correlation *r* = 0.97), with the exception of centromeric regions, where the histone H3 variant Cse4^CENP-A^ replaced histone H3 (Fig. 1A and C; see also Fig. S2A). Furthermore, RNAPII occupancy showed a positive correlation with gene expression levels (Pearson correlation *r* = 0.72) (Fig. 1B).

10.1128/mBio.01376-19.2 FIG S2 (A) Fold enrichment (log_2_) of histone H3 relative to unmodified H4 across the centromeric and pericentromeric regions of all 8 chromosomes in C. albicans. The CenpA enrichment profile is shown for comparison. The blue bars indicate statistically significantly depleted regions for histone H3. (B) Histogram of RNA-seq counts (log_2_) in WT cells across all protein-coding genes, according to assembly 22. Genes were grouped into four sets organized by expression level as follows: no expression (green, log_2_ counts of <2, *n* = 416), low expression (blue, log_2_ counts of 2 to 4, *n* = 1,369), medium expression (red, log_2_ counts of 4 to 6, *n* = 3,570) and high expression (yellow, log_2_ counts of >6, *n* = 983). Download FIG S2, TIF file, 0.9 MB. Copyright © 2019 Price et al. 2019 Price et al. This content is distributed under the terms of the Creative Commons Attribution 4.0 International license.

10.1128/mBio.01376-19.9 TABLE S2 Sequencing and coverage information of ChIP-seq and RNA-seq data sets. Download Table S2, XLSX file, 0.02 MB. Copyright © 2019 Price et al. 2019 Price et al. This content is distributed under the terms of the Creative Commons Attribution 4.0 International license.

### H3K4me^3^, H3K9Ac, and H4K16Ac mark C. albicans active genes.

To delineate the chromatin signature of protein-coding genes, enrichment profiles corresponding to each histone modification were compared to the histone H4 profile. Differential enrichment testing using DESeq2 allowed the identification of regions with statistically significant enrichment or depletion for particular histone marks compared to histone H4. We annotated these loci by proximity to annotated protein-coding genes and noncoding RNAs (25–27). For RNAPII, aligned reads from ChIP (Ip) samples were normalized to aligned reads from the matching input (I) sample.

**FIG 2:**
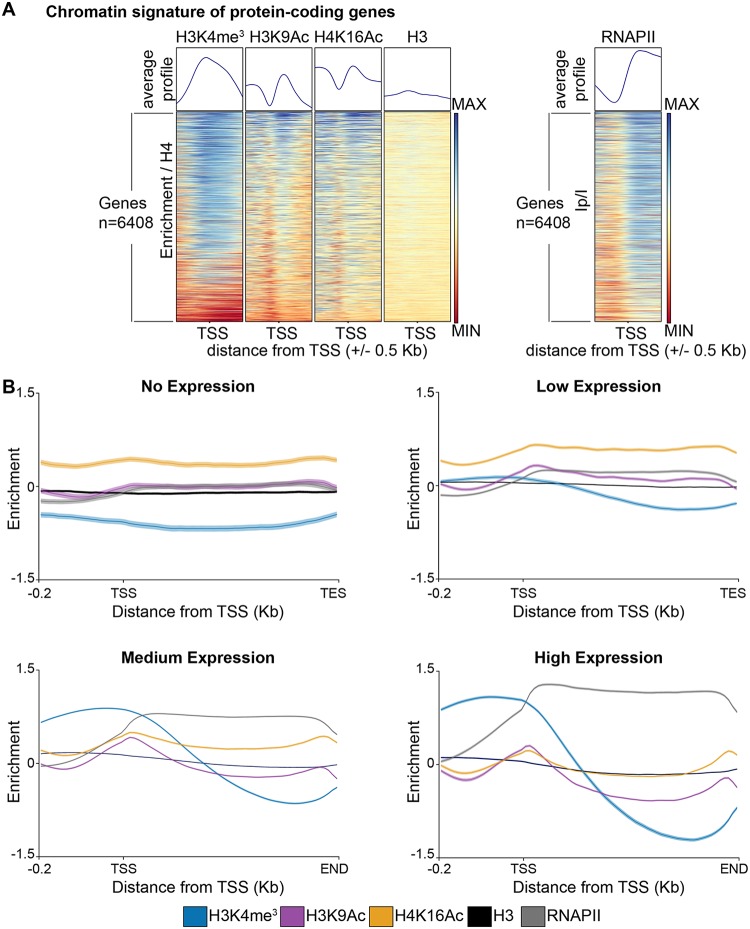
C. albicans chromatin modifications mirror their transcriptional state. (A) Chromatin signature of C. albicans genes (*n* = 6,408). Average profiles and heat maps of histone modification signatures around the Transcriptional Start Sites (TSS) of genes. The relative fold enrichment (log_2_) levels for each histone modification normalized to unmodified histone H4 or for aligned reads of immunoprecipitated (IP) sample normalized to aligned reads of input (I) sample (for RNAPII ChIP-seq) are displayed within a region spanning ±0.5 kb around the TSS. The blue-to-red color gradient indicates high to low levels of enrichment in the corresponding region. MIN (minimum), −1.5 log_2_; MAX (maximum), +1.5 log_2_. (B) Average profiles of histone modifications and RNAPII occupancy across gene sets with different expression levels (no [*n* = 416], low [*n* = 1,369], medium [*n* = 3,570], and high [*n* = 983] expression). For each histone modification, the fold enrichment (log_2_) relative to unmodified H4 is shown. For RNAPII, enrichment (log_2_) levels are shown as IP/I data (aligned reads of immunoprecipitated [IP] sample normalized to aligned reads of input [I] sample). TES, transcriptional end site.

Metagene analyses demonstrated that, as expected, RNAPII was enriched across all gene bodies whereas unmodified histone H3 was not significantly enriched or depleted relative to unmodified histone H4. In contrast, H3K4me^3^ and H3K9Ac were more prominent at the transcriptional start site (TSS [the first nucleotide of each open reading frame]) and 5′ regions of genes, and H4K16Ac was enriched across gene bodies (Fig. 2A).

To further explore the relationship between chromatin modifications and gene transcriptional states, we grouped all genes into four sets based on expression level (no expression, low expression, medium expression, and high expression) as revealed by RNA-seq analysis (Fig. S2B). Enrichment profile plots of the levels of histone modifications for each of these gene sets demonstrated that H3K4me^3^, H3K9Ac, and H4K16Ac levels were very low at genes with low transcription rates. Levels of all modifications increased with increased gene expression, reaching a maximum at highly transcribed genes (Fig. 2B). Therefore, in C. albicans, H3K4me^3^, H3K9Ac, and H4K16Ac correlate with gene transcription; H3K4me^3^ and H3K9Ac are more highly enriched at the 5′ end of a gene and H4K16Ac at the gene bodies.

### The chromatin state of the C. albicans repetitive genome.

Having determined the chromatin marks associated with C. albicans coding genes, we analyzed the chromatin state of the C. albicans repetitive genome, focusing on the major classes of DNA repeats: subtelomeric regions, the rDNA locus, MRSs, and transposable elements (LTR and non-LTR retrotransposons). Sequence analysis of these elements can be problematic because of incomplete sequencing and because of their repetitive nature (25, 29). To estimate the chromatin modification state of these loci, we adopted a method previously applied to S. cerevisiae repeats and assumed that all repeats contribute equally to read depth (43). Consequently, reads that could not be uniquely mapped to one location were randomly assigned to copies of that repeat.

**FIG 3:**
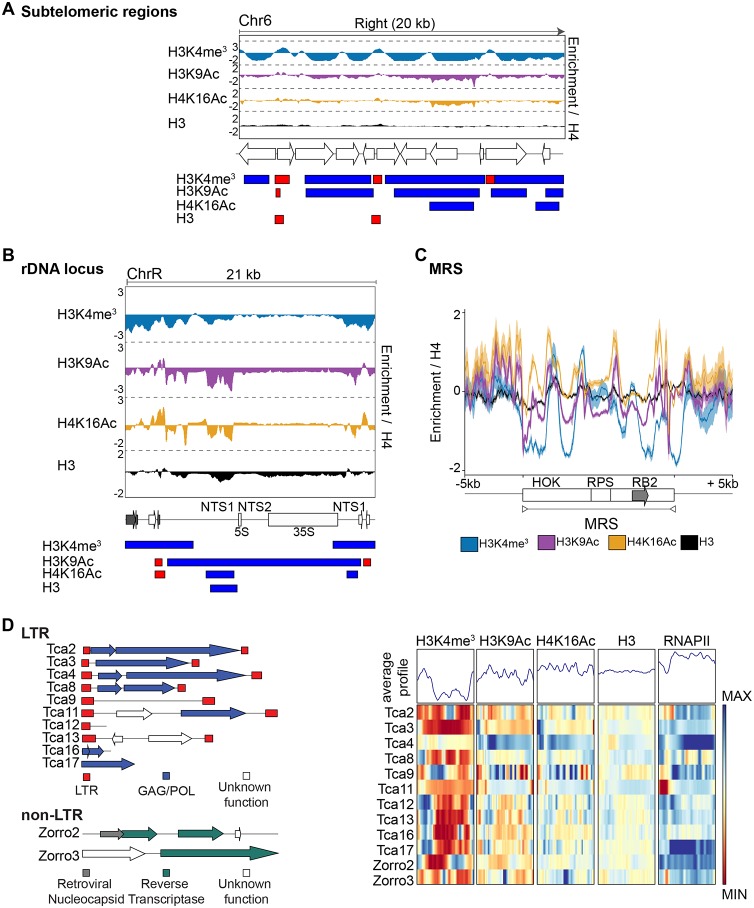
Chromatin signature of C. albicans repetitive elements. (A) (Top) Fold enrichment (log_2_) of H3K4me^3^_,_ H3K9Ac, H4K16Ac, and H3 relative to unmodified H4 across the 20-kb right terminal region of chromosome 6 (Chr6). (Middle) Diagram of coding genes found at these regions, according to assembly 22. (Bottom) Diagram depicting statistically significantly enriched (red) or depleted (blue) domains for each histone modification. (B) (Top) Fold enrichment (log_2_) of H3K4me3_,_ H3K9Ac, H4K16Ac, and H3 relative to unmodified H4 at the rDNA locus and flanking regions (ChrR). (Middle) Diagram of coding genes (white) and ncRNAs (gray) found at this region, according to assembly 22. (Bottom) Diagram depicting statistically significantly enriched (red) or depleted (blue) domains for each histone modification. (C) Average profiles of histone modifications at MRSs and at upstream and downstream sequences. The gray arrow indicates the location of the FGR gene. For each histone modification, the fold enrichment (log_2_) relative to unmodified H4 is shown. (D) (Left) Diagrams of the structure of the C. albicans LTR and non-LTR retrotransposons. (Right) Chromatin signature of LTR and non-LTR retrotransposons. Average profiles and heat maps of histone modification signatures across each sequence are shown. The relative fold enrichment (log_2_) levels for each histone modification normalized to unmodified histone H4 or for aligned reads of immunoprecipitated (IP) sample normalized to aligned reads of input (I) sample (for RNAPII ChIP-seq) are displayed. The blue-to-red color gradient indicates high to low levels of enrichment in the corresponding region. MIN (minimum), −1.5 log_2_; MAX (maximum), +1.5 log_2_.

To investigate the chromatin state associated with the 16 subtelomeric regions in C. albicans, we analyzed the ChIP-seq data sets in the 20-kb terminal regions of each chromosome arm. At these locations, occupancy of unmodified histone H3 was similar to histone H4 occupancy (Fig. 3A; see also Fig. S3). In contrast, we detected large domains of chromatin that were hypomethylated on H3K4 and hypoacetylated on H3K9 and H4K16 (Fig. 3A; see also Fig. S3). However, the states of H3K4 methylation and H3K9/H4K16 acetylation of subtelomeres were not uniform, as patches of high levels of H3K4me^3^, H3K9Ac, and H4K16Ac were detected within each subtelomere (Fig. 3A; see also Fig. S3).

10.1128/mBio.01376-19.3 FIG S3 (Top) Fold enrichment (log_2_) of H3K4me^3^_,_ H3K9Ac, H4K16Ac, γH2A, and H3 relative to unmodified H4 across the 20-kb left and right terminal regions of each of the 8 C. albicans chromosomes. (Middle) Diagrams of coding genes found at these regions (grey: TLO), according to assembly 22. (Bottom) Diagram depicting statistically significantly enriched (red) or depleted (blue) domains for each histone modification. Download FIG S3, TIF file, 1.1 MB. Copyright © 2019 Price et al. 2019 Price et al. This content is distributed under the terms of the Creative Commons Attribution 4.0 International license.

Analysis of chromatin modifications associated with the rDNA locus demonstrated that the NTS1 and NTS2 regions are assembled into a chromatin structure resembling heterochromatin, where nucleosomes are hypomethylated on H3K4 and hypoacetylated on H3K9 and H4K16 (Fig. 3B), consistent with our data published previously demonstrating that these regions are assembled into transcriptionally silent heterochromatin (44).

This analysis also revealed that MRSs and retrotransposons (LTR and non-LTR) are associated with chromatin, which is largely hypomethylated on H3K4 (Fig. 3C and D). In contrast, the H3K9Ac and H4K16Ac levels were similar to histone H4 levels (Fig. 3C and D). Therefore, different C. albicans repetitive elements are associated with distinct chromatin states. Repetitive regions are more likely to be hypomethylated on H3K4 but not hypoacetylated on H3K9 and H4K16.

### γH2A is enriched at convergent genes and in proximity to DNA replication origins.

Having established that gene-rich and repeat-rich regions of the C. albicans genome are marked by different chromatin modifications depending on their transcriptional state (Fig. 2; see also Fig. 3), we sought to systematically map the genome-wide profile of γH2A (γ-sites) in cycling, undamaged cells, as this is a useful method to identify recombination-prone unstable sites (14). Genome-wide ChIP-seq analysis of γH2A identified 171 γ-sites where γH2A was enriched compared to histone H4. However, γH2A enrichment across the C. albicans genome is less pronounced than that seen with the other histone modifications analyzed (see Data Set S1 in the supplemental material). C. albicans γ-sites are different from the γH2A domain caused by irrecoverable DSBs, as γ-sites generally have a single peak of enrichment and are shorter (average length, 850 bp) than the 50-kb γH2A domains surrounding DSBs (45).

10.1128/mBio.01376-19.10 DATA SET S1 Tables of significant outcomes from statistical analyses. (A – E) Regions of enriched/depleted histone modifications compared to unmodified histone H4 in WT cells, as determined by DESeq2. (F – H) Regions of enriched/depleted histone modifications in mutant strains compared to the WT cells, as determined by two sample t-test. (I, J) RNA-seq analysis in mutant strains compared to the WT cells, as determined by DESeq2. Download Data Set S1, XLSX file, 2.2 MB. Copyright © 2019 Price et al. 2019 Price et al. This content is distributed under the terms of the Creative Commons Attribution 4.0 International license.

**FIG 4:**
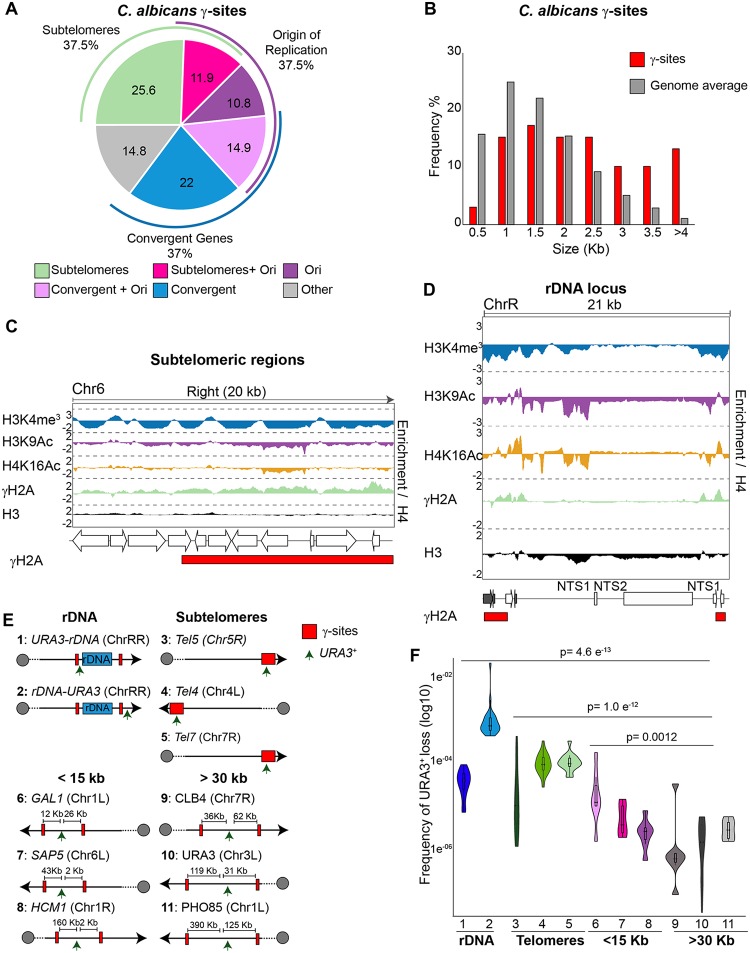
Identification of C. albicans γ-sites (*n* = 171). (A) Locations and frequencies of γ-sites throughout the C. albicans genome. (B) γ-sites map to longer genes. The histogram shows the gene lengths of γ-sites (red) compared to the genome average (gray). (C) (Top) Fold enrichment (log_2_) of H3K4me^3^_,_ H3K9Ac, H4K16Ac, γH2A, and H3 relative to unmodified H4 across the 20-kb right terminal region of chromosome 6 (Chr6). (Middle) Diagram of coding genes found at these regions, according to assembly 22. (Bottom) Diagram depicting statistically significantly enriched (red) domains for γH2A. (D) (Top) Fold enrichment (log_2_) of H3K4me3_,_ H3K9Ac, H4K16Ac, γH2A, and H3 relative to unmodified H4 at the rDNA locus and flanking regions (ChrR). (Middle) Diagram of coding genes (white) and ncRNAs (gray) found at this region, according to assembly 22. (Bottom) Diagram depicting statistically significantly enriched (red) domains for γH2A. (E) Schematic of strains (1 to 11) used to measure genome instability in relation to γ-sites (red box). The positions of *URA3* insertions (one per strain) are indicated (arrow). (F) Frequency of URA3 loss in strains (1 to 11) containing URA3 heterozygous insertions.

Analysis of γ-sites indicates that they are present with three classes of genomic loci: (i) subtelomeric regions, (ii) longer genes, which are often convergent; and (iii) origins of replication (46) (Fig. 4A and B).

We detected statistically significant γH2A enrichment at 13/16 subtelomeres (Fig. 4C; see also Fig. S3). We suspect that the absence of γ-sites at chromosome RR (ChrRR), Chr1R, and Chr7L subtelomeric regions is due to incomplete genome assembly (25, 29). Subtelomeric γH2A enrichment levels were not uniform but were present at distinct peaks within subtelomeres, which largely associated with hypoacetylated and hypomethylated chromatin (Fig. 4C; see also Fig. S3). At convergent genes, γH2A enrichment was detected at both the gene bodies and the intergenic regions. Two γ-sites at convergently transcribed genes surrounded the rDNA locus (Fig. 4D). γH2A enrichment was not a feature of all of the repetitive elements, as we did not detect any statistically significant enrichment of γH2A at either MRSs or retrotransposons.

No correlation was detected between gene expression levels and γH2A occupancy. Although we did not observe any correlation between γ-sites and histone H3 occupancy (Pearson correlation *r* = 0.062), γ-sites are more likely to mark genomic regions that are acetylated on H4K16 and H3K9 (Pearson correlation *r* = 0.461 and 0.276, respectively). We also detected a weak negative correlation between γH2A occupancy and H3K4me^3^ occupancy (Fig. S4).

10.1128/mBio.01376-19.4 FIG S4 Chromatin signature of γ-sites throughout the C. albicans genome. Average profiles and heat maps of histone modification signatures at γ-sites are shown. The relative fold enrichment (log_2_) for each histone modification normalized to unmodified histone H4 is displayed within a region spanning ±2 kb around the γH2A peak summits. The blue-to-red color gradient indicates high to low levels of enrichment in the corresponding region. MIN (minimum), −1.5 log_2_; MAX (maximum), +1.5 log_2_. Download FIG S4, TIF file, 1.5 MB. Copyright © 2019 Price et al. 2019 Price et al. This content is distributed under the terms of the Creative Commons Attribution 4.0 International license.

The γ-sites mapped in this study are candidate fragile sites that are more prone to intrinsic DNA damage and genome instability. Analysis of the frequency of a heterozygous *URA3^+^* marker gene integrated at different genomic locations (47, 48) revealed that *URA3* loss was more frequent when the gene is integrated in proximity to γ-sites (rDNA locus and subtelomeres and less than 15 kb away from a γ-site[s]) than when integrated far away (>30 Kb) from γ-sites (Fig. 4E and F). These results support the hypothesis that γ-sites are recombination-prone genomic sites.

We therefore concluded that γH2A marks discrete genomic regions in cycling C. albicans cells and that these loci are potential unstable sites.

### HDAC Sir2 governs the hypoacetylated state associated with C. albicans rDNA locus and subtelomeric regions.

We previously found that Sir2 HDAC maintains low level of H3K9Ac associated with the NTS regions of the rDNA locus in C. albicans (44). To assess the role of Sir2 in maintaining acetylation levels across the C. albicans genome, we performed H3K9Ac and H4K16Ac ChIP-seq analyses in WT and *sir2Δ*/*Δ* strains.

**FIG 5:**
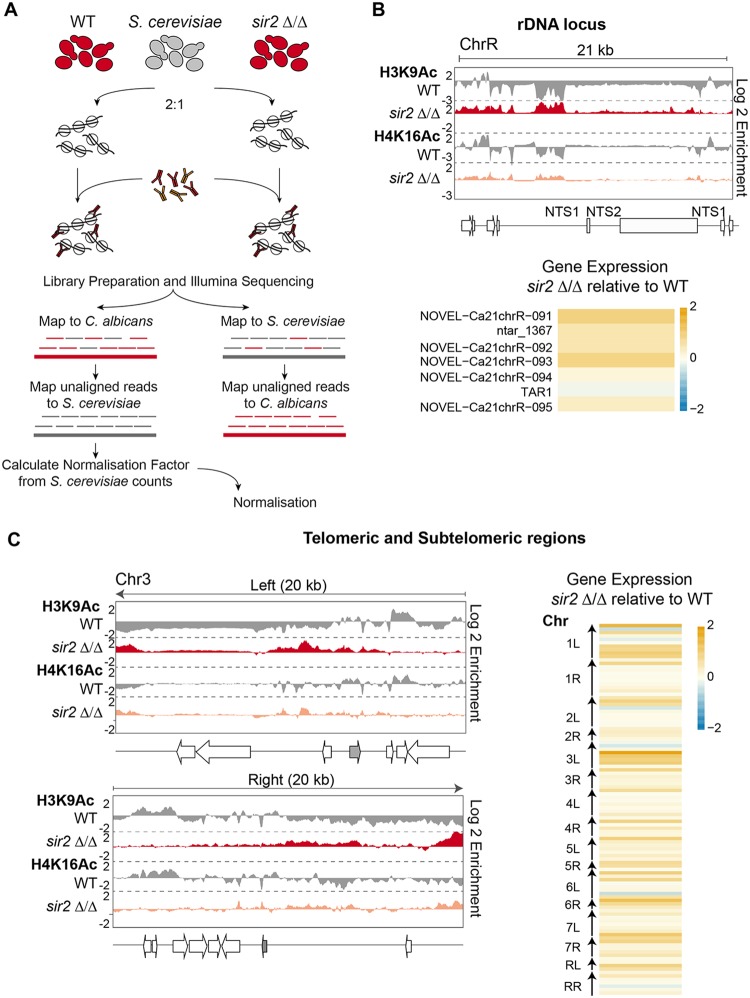
The Sir2 HDAC controls the chromatin state of subtelomeres and the rDNA locus. (A) Schematic of the quantitative ChIP-seq experimental and analytical workflow. (B) (Top) Fold enrichment (log_2_) of H3K9Ac and H4K16Ac relative to unmodified H4 in WT cells, and relative to the WT in *sir2Δ/Δ* cells, across the rDNA loci of chromosome R (ChrR). (Middle) Diagram of transcripts found at this region, according to assembly 22. (Bottom) Heat map depicting changes in gene and ncRNA expression levels across the rDNA region in *sir2Δ/Δ* cells relative to the WT. The yellow-to-blue color gradient indicates high to low levels of expression. MIN (minimum), −2 log_2_; MAX (maximum), +2 log_2_. (C) (Left) Fold enrichment (log_2_) of H3K9Ac and H4K16Ac relative to unmodified H4 in WT cells, and relative to the WT in *sir2Δ*/*Δ* cells, across the 20-kb left and right terminal regions of chromosome 3 (Chr3). Diagrams of coding genes (*TLO*: gray) found at these regions, according to assembly 22, are shown at the bottom. (Right) Heat map depicting changes in gene and ncRNA expression levels in *sir2Δ*/*Δ* cells relative to the WT at the 10-kb terminal regions of all C. albicans chromosomes. The yellow-to-blue color gradient indicates high to low levels of expression. MIN (minimum), −2 log_2_; MAX (maximum), +2 log_2_.

Traditional ChIP-seq experiments are not inherently quantitative. They allow comparisons of levels of protein occupancy at different positions within a genome but do not allow direct comparisons between samples derived from different strains (49–51). To overcome this issue, we adapted a quantitative ChIP-seq (q-ChIP-seq) methodology for use in C. albicans (49–51). To this end, we spiked in a single calibration sample from S. cerevisiae at the time of fixation of both WT and *sir2Δ*/*Δ* cells (Fig. 5A). The S. cerevisiae genome is a useful exogenous reference for C. albicans cells because its genome is well studied and has a high-quality sequence assembly (52). Moreover, reads originating from C. albicans or S. cerevisiae can be easily separated at the analysis level and our experiments revealed that less than 2% of the total number of reads cannot be uniquely mapped (Table S2). Finally, histone proteins are well conserved between C. albicans and S. cerevisiae (Fig. S1) and, therefore, the same histone antibody immunoprecipitates C. albicans and S. cerevisiae chromatin with similar efficiency levels.

The q-ChIP-seq results demonstrated that deletion of SIR2 leads to a modest and variable increase of H3K9Ac and H4K16Ac levels. Only two regions of the C. albicans genome displayed significantly increased H3K9Ac and H4K16Ac levels in *sir2Δ*/*Δ* mutant cells: subtelomeric regions and the NTS region of the rDNA locus (Fig. 5B and C; see also Fig. S5 and Data Set S1). Deletion of *SIR2* did not lead to increased histone acetylation levels at euchromatic regions or at other repetitive elements such as MRSs and retrotransposons. In agreement with these findings, the 83% of gene expression changes observed in *sir2Δ*/*Δ* cells occurred at the rDNA locus and subtelomeric regions (44) (Fig. 5B and C; see also Data Set S1). Thus, it appears that C. albicans Sir2 acts exclusively at two genomic regions: the rDNA locus and subtelomeric regions. These results are consistent with the hypothesis that Sir2-mediated histone deacetylation represses gene expression at these locations.

10.1128/mBio.01376-19.5 FIG S5 (Top) Fold enrichment (log_2_) of H3K9Ac and H4K16Ac relative to unmodified H4 in WT cells, and relative to the WT in *sir2 Δ/Δ* cells, across the 20 kb left and right terminal regions of each of the 8 C. albicans chromosomes. (Bottom) Diagrams of coding genes found at these regions (grey: TLO), according to assembly 22. Download FIG S5, TIF file, 0.9 MB. Copyright © 2019 Price et al. 2019 Price et al. This content is distributed under the terms of the Creative Commons Attribution 4.0 International license.

### Set1-dependent methylation of H3K4 affects gene expression differentially at different repeats.

**FIG 6:**
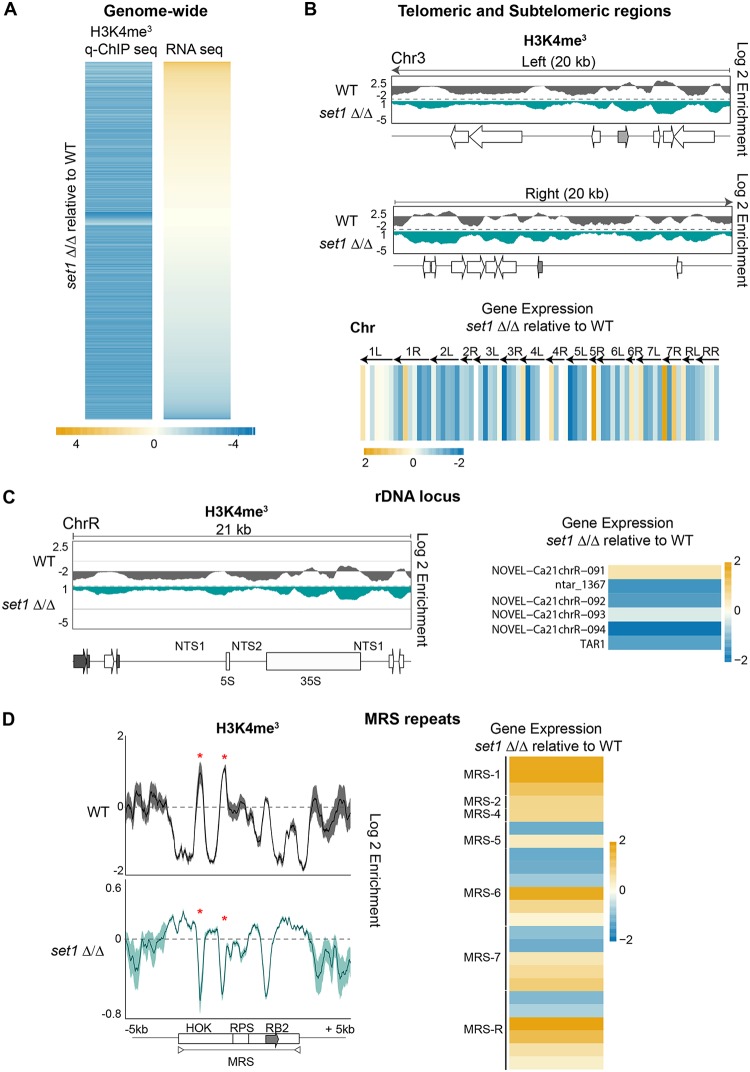
Chromatin and gene expression changes of *set1*Δ/Δ strain. (A) Heat map depicting changes in expression of genes and ncRNAs associated with statistically significant changes in H3K4me^3^ enrichment in *set1Δ*/*Δ* cells relative to the WT cells. The yellow-to-blue color gradient indicates high to low levels of enrichment/expression. MIN (minimum), −4 log_2_; MAX (maximum), +4 log_2_. (B) (Top) Fold enrichment (log_2_) of H3K4me^3^ relative to unmodified H4 in WT cells, and relative to the WT in *set1Δ*/*Δ* cells, across the 20-kb left and right terminal regions of chromosome 3 (Chr3). Diagrams of coding genes (*TLO*: gray) found at these regions, according to assembly 22, are shown below the fold enrichment data. (Bottom) Heat map depicting changes in gene and ncRNA expression levels in *set1Δ*/*Δ* cells relative to the WT at the 10-kb terminal regions of all C. albicans chromosomes. The yellow-to-blue color gradient indicates high to low levels of expression. MIN (minimum), −2 log_2_; MAX (maximum), +2 log_2_. (C) (Left) Fold enrichment (log_2_) of H3K4me^3^ relative to unmodified H4 in WT cells, and relative to the WT in *set1Δ*/*Δ* cells, across the rDNA loci of chromosome R (ChrR). Diagrams of coding genes and ncRNAs (gray) found at this region, according to assembly 22, are shown at the bottom. (Right) Heat map depicting changes in gene and ncRNA expression levels across the rDNA region in *set1Δ*/*Δ* cells relative to the WT. The yellow-to-blue color gradient indicates high to low levels of expression. MIN (minimum), −2 log_2_; MAX (maximum), +2 log_2_. (D) (Left) Profiles of fold enrichment (log_2_) of H3K4me^3^ relative to unmodified H4 in WT cells, and relative to the WT in *set1Δ*/*Δ* cells averaged across the MRSs, and the upstream and downstream sequences. The gray arrow indicates the location of the FGR gene. (Right) Heat map depicting changes in gene and ncRNA expression levels across all of the MRS regions in *set1Δ*/*Δ* cells relative to the WT. The yellow-to-blue color gradient indicates high to low levels of expression. MIN (minimum), −2 log_2_; MAX (maximum), +2 log_2_.

C. albicans repetitive elements are associated with chromatin that is hypomethylated on H3K4, and yet, pockets of H3K4me^3^ are detected at these regions, indicating that H3K4 methylation is not completely ablated (Fig. 3). In S. cerevisiae and S. pombe, H3K4 methyltransferase Set1 has been implicated in both gene repression and gene activation (53–58). S. cerevisiae Set1 also maintains the transcriptional silencing associated with heterochromatic regions such as the telomeres and the rDNA locus (53–58). To gain insights into the role of C. albicans Set1, we performed H3K4me^3^ q-ChIP-seq and RNA-seq analyses of WT and *set1Δ*/*Δ* strains. Strikingly, in the *set1Δ*/*Δ* strain, 7,186 loci across the genome showed a statistically significant reduction of H3K4me^3^ levels in the *set1Δ*/*Δ* strain compared to the WT strain (Fig. 6A; see also Data Set S1). Thus, C. albicans Set1 clearly plays a major role in maintaining chromatin structure. RNA-seq analysis revealed that Set1 regulates gene expression both positively and negatively, as genes with a pattern of reduced H3K4me^3^ levels were either upregulated (1,200 genes/noncoding RNAs [ncRNAs]) or downregulated (1,550 genes/ncRNAs) in strain *set1Δ*/*Δ* compared to the WT (Fig. 6A; see also Data Set S1). Analyses of the H3K4me^3^ pattern and gene expression levels associated with repetitive elements revealed that Set1 has distinct roles at different repeats. At subtelomeric regions and the rDNA locus, strain *set1Δ*/*Δ* had reduced H3K4me^3^ levels that were accompanied by the downregulation of nearby genes (Fig. 6B and C; see also Fig. S6). In contrast, at MRSs in strain *set1Δ*/*Δ*, the reduced H3K4me^3^ pattern was associated with increased expression of coding and noncoding RNAs originating from MRSs (Fig. 6D). Finally, at transposons in strain *set1Δ*/*Δ*, there was no significant effect on the expression of retrotransposon-associated coding and noncoding RNAs.

10.1128/mBio.01376-19.6 FIG S6 (Top) Fold enrichment (log_2_) of H3K4me^3^ relative to unmodified H4 in WT cells, and relative to the WT in *set1 Δ/Δ* cells, across the 20 kb left and right terminal regions of each of the 8 C. albicans chromosomes. (Bottom) Diagrams of coding genes found at these regions (grey: TLO), according to assembly 22. Download FIG S6, TIF file, 0.8 MB. Copyright © 2019 Price et al. 2019 Price et al. This content is distributed under the terms of the Creative Commons Attribution 4.0 International license.

We conclude that Set1 has major effects on chromatin structure and gene expression across the genome in C. albicans. Importantly, strain *set1Δ*/*Δ* showed decreased H3K4 methylation across all repetitive elements, and yet, Set1 influenced gene expression differentially at each repetitive element.

## DISCUSSION

Here, we performed the first comprehensive chromatin profiling of histone modifications associated with the C. albicans genome, where we specifically focused on the similarities and differences at repetitive genomic regions. Furthermore, we present the first C. albicans quantitative ChIP-seq analysis performed to delineate the roles of chromatin modifiers Sir2 and Set1.

Our first objective was to obtain “proof-of-concept” epigenomic maps of chromatin modifications associated with gene-rich regions of the C. albicans genome. A robust histone modification profile relies on (i) the use of antibodies that recognize modified histones with high specificity and (ii) the use of appropriate biological controls. The specificity of antibodies used in this study has been tested using S. pombe or S. cerevisiae histone mutants lacking the modifiable amino acid (H3K9, H4K16, and H2AS129) (14, 59). To distinguish between nucleosome occupancy and depletion/enrichment of specific histone modifications, ChIP-seq was also performed with antibodies that recognize unmodified histone H3 and H4. This is an important control that should be included in all studies designed for genome-wide analysis of chromatin modifications.

The results confirm that the experimental approach is valid and that C. albicans active chromatin conforms to the histone modification pattern reported in other organisms: active genes, associated with high levels of RNA Pol II, are assembled into canonical euchromatin, with H3K4me^3^ and H3K9Ac associated with promoters and H4K16Ac enriched at gene bodies. Thus, the specific histone modification pattern predictive of active transcription in other organisms is conserved in C. albicans.

We next asked about the repeated sequences in the C. albicans genome that are more likely to have properties of heterochromatin. The chromatin in NTS regions of the rDNA locus and in the subtelomeric regions resembles the heterochromatic structure of the budding yeast S. cerevisiae, which lacks H3K9me/H3K27me systems (43). This is consistent with the ability of the rDNA locus and subtelomeric regions to silence embedded marker genes, a hallmark of heterochromatic regions (44). In stark contrast, C. albicans retrotransposons and MRSs are assembled into a distinct chromatin state; nucleosomes are hypomethylated on H3K4me^3^ and also acetylated on H3K9 and H4K16. In the host, transposons and MRSs are recombination hotspots as they are known sites of translocations (32, 37, 38). Given the key role of chromatin in regulating genome accessibility and stability, it will be important to investigate whether the chromatin packaging of MRSs and transposons influences recombination at these sites.

Since γH2A is an important hallmark of recombination-prone sites (20), we investigated the localization of γH2A across the C. albicans genome. We found that γH2A is enriched at telomeres and at the rDNA locus, heterochromatic regions assembled into hypoacetylated chromatin that is also hypomethylated on H3K4. This is similar to observations in other fungal organisms where γH2A decorates heterochromatic regions (14–17). In contrast, γH2A was not enriched at MRSs and transposable elements. This is surprising because, in the host, MRSs are recombination hotspots (32, 38) and therefore are regions where DSBs are expected to accumulate. C. albicans genome instability is increased under conditions of host-relevant stresses (47, 48). Therefore, we propose that the recombination potential of MRS is unlocked following exposure to the specific host niche stresses.

Finally, we detected γ-sites located in proximity to origins of replication or convergent genes that are often long. DNA replication origins are known replication fork barriers in many organisms, and read-through transcription of convergent genes can also cause genome instability as a consequence of, for example, R-loop formation (60). We propose that the γ-sites identified in this study represent novel recombination-prone unstable sites of the C. albicans genome. In support of this hypothesis, we find that genome instability is higher in proximity to γ-sites.

We present the first quantitative ChIP-seq analysis in C. albicans that delineates the roles of histone-modifying enzymes Sir2 and Set1. We found that Sir2 maintains the hypoacetylated state of heterochromatic regions associated with the rDNA locus and subtelomeric regions. Sir2 deacetylation at these loci is associated with gene repression, as shown by RNA-seq analysis. In contrast, deletion of Sir2 does not lead to increased histone acetylation and gene expression at other genomic regions. Either of two possible scenarios could explain these findings: (i) Sir2 is specifically targeted to subtelomeres and the rDNA locus or (ii) other histone deacetylases act redundantly with Sir2 in regulating hypoacetylation and gene expression at other genomic locations. In support of the second hypothesis, note that the C. albicans genome encodes 9 histone deacetylases and that functional redundancy among these enzymes has been reported in C. albicans and other organisms (61–63). Either way, Sir2 largely affects the telomere regions, consistent with the Sir2-dependent stochastic silencing of the *TLO* genes (64).

HMT Set1 is a major determinant of C. albicans H3K4me^3^ expression. Reduced H3K4me^3^ levels are associated with extensive gene expression changes, demonstrating that Set1 is the major C. albicans H3K4 methyltransferase. It is particularly intriguing that deletion of *SET1* leads to decreased H3K4me^3^ levels at all known repeats and yet its effects on gene expression can occur in both directions. Indeed, we demonstrated that Set1 represses gene expression at the rDNA locus and subtelomeric regions while it activates gene expression at MRSs.

In this report, we present the first epigenomic map of histone modifications associated with the C. albicans genome. We observed patterns of histone modifications at coding regions and repetitive regions. We also identified unexpected patterns of histone marks at MRS and retrotransposons and also characterized the roles of Sir2 and Set1 in C. albicans. Given the key role of chromatin in regulating C. albicans biology, the data generated in this study provide an invaluable resource for better understanding of this important human fungal pathogen.

## MATERIALS AND METHODS

### Yeast growth and manipulation.

Strains used in this study are listed in Table S1 in the supplemental material. Yeast were cultured in YPAD broth containing 1% yeast extract, 2% peptone, 2% dextrose, 0.1 mg/ml adenine, and 0.08 mg/ml uridine at 30°C.

10.1128/mBio.01376-19.8 TABLE S1 Strains used in this study. Download Table S1, DOCX file, 0.02 MB. Copyright © 2019 Price et al. 2019 Price et al. This content is distributed under the terms of the Creative Commons Attribution 4.0 International license.

### Antibody information.

The antibodies used in this study were as follows: anti-H2AS129p (Millipore; catalog no. 07-745-I), anti-H3 (Abcam; catalog no. ab1791), anti-H4 (Millipore; catalog no. 05-858), anti-H3K4me3 (Active Motif; catalog no. 39159), anti-H3K9ac (Active Motif; catalog no. 39137), anti-H4K16ac (Active Motif; catalog no. 39167), and anti-RNA polymerase II (BioLegend; catalog no. 664903).

### ChIP-seq.

Chromatin immunoprecipitation with massively parallel deep sequencing (ChIP-seq) was performed as follows. A 5-ml volume of an overnight culture grown in YPAD was diluted into fresh YPAD and grown until the exponential phase was reached (optical density at 600 nm [OD_600_] = 0.6 to 0.8). Cells (OD_600_ = 20) were fixed with 1% formaldehyde (Sigma) for 15 min at room temperature. Reactions were quenched by the addition of glycine to reach a final concentration of 125 mM. Cells were lysed using acid-washed glass beads (Sigma) and a DisruptorGenie (Scientific Industries) for four cycles of 30 min at 4°C with 5 min on ice between cycles. Chromatin was sheared to 200 to 500 bp using a BioRuptor sonicator (Diagenode) for a total of 20 min (30 s on, 30 s off cycle) at 4°C. Immunoprecipitation was performed overnight at 4°C using 2 μl of the appropriate antibody and 25 μl of protein G magnetic Dynabeads (Invitrogen). ChIP DNA was eluted, and cross-links were reversed at 65°C in the presence of 1% SDS. All samples were then treated with RNase A and proteinase K before being purified by phenol/chloroform extraction and ethanol precipitation. Libraries were prepared and sequenced as 50-bp single-end reads on an Illumina HiSeq2000 platform by the Genomics Core Facility at EMBL (Heidelberg, Germany). All ChIP-seq experiments were carried out in biological duplicate.

### q-ChIP-seq.

Quantitative ChIP-seq (q-ChIP-seq) was performed similarly to the ChIP-seq method, except 5 ml of an overnight culture of S. cerevisiae reference strain BY4741 was grown alongside C. albicans in YPAD. These cultures were then diluted into fresh YPAD and grown until the exponential phase was reached (OD_600_ = 0.6 to 0.8). C. albicans cells were combined with S. cerevisiae cells (OD_600_ = 10) and then fixed with 1% formaldehyde (Sigma) for 15 min at room temperature. After the cells had been fixed, the q-ChIP-seq sample was processed as a single ChIP-seq sample throughout the experiment until completion of DNA sequencing. All q-ChIP-seq experiments were carried out in biological duplicate.

### RNA-seq.

RNA was extracted from exponential cultures (OD_600_ = 0.6 to 0.8) using a yeast RNA extraction kit (E.Z.N.A. isolation kit RNA yeast; Omega Bio-Tek) following the manufacturer’s instructions. RNA quality was checked by electrophoresis under denaturing conditions in 1% agarose–1× HEPES–6% formaldehyde (Sigma). RNA concentrations were measured using a NanoDrop ND-1000 spectrophotometer. Strand-specific cDNA Illumina barcoded libraries were generated from 1 μg of total RNA and sequenced as 50-bp single-end reads using an Illumina HiSeq2000 sequencer by the Genomics Core Facility at EMBL (Heidelberg, Germany). All RNA-seq experiments were carried out in biological duplicate.

### Genome instability assay.

Strains were first streaked on –Uri media to ensure the selection of cells carrying the *URA3^+^* marker gene. Parallel liquid cultures (7 to 15) were pregrown overnight from independent single colonies. Each culture was diluted to a concentration of 100 cells/μl and grown for nine generations (18 h). Cells were plated on synthetic complete (SC) plates containing 1 mg/ml 5-FOA (5-fluorotic acid; Sigma) and on nonselective SC plates and grown at 30°C. Colonies were counted after 2 days of growth, and the frequency of 5‐FOA‐resistant (5‐FOA^r^) colony appearance (also referred to below as the frequency of *URA3* loss) was calculated using the formula *F* = *m*/*M*, where *m* represents the median number of colonies obtained on 5‐FOA medium corrected by the dilution factor used and the fraction of culture plated and *M* the average number of colonies obtained on YPD corrected by the dilution factor used and the fraction of culture plated (65). Statistical differences between results from samples were calculated using the Kruskal-Wallis test and the Mann-Whitney U test for *post hoc* analysis. Statistical analysis was performed and violin plots were generated using R Studio.

### Analysis of high-throughput sequencing. (i) ChIP-seq analysis.

Illumina reads were mapped using Bowtie2 (66) to a custom haploid version of assembly 22 of the C. albicans genome (Table S2). Reads that mapped to repeated sequences were randomly assigned to copies of that repeat, allowing for an estimation of enrichment at the repetitive elements of the genome. Peak calling was performed using MACS2 (67) and the default settings, except that no model was used with all reads extended to 250 bp. MACS2 was run separately on the two biological replicates for each ChIP-seq sample. For each sample analyzed with MACS2, the IP sample represented the “treatment” and the input sample represented the “control.” We defined peaks as reproducible if they were called in both data sets. Read counts within peak intervals were generated using featureCounts (68). For each interval, biological duplicate counts corresponding to each histone modification and unmodified histone H4 samples were compared using DESeq2, with an adjusted *P* value threshold of <0.05 being used to identify significant differences. Replicates were compared by generating a raw alignment coverage track and performing a Pearson correlation analysis using the multiBamSummary and plotCorrelation tools as part of the deepTools2 package (Fig. S7) (69). Genome coverage tracks were made using the pileup function of MACS2 (67), and data representing tracks from biological replicates were averaged after the replicates were deemed to be sufficiently correlative (*r*, >0.9). For each coverage track, reads per million (RPM) were calculated. The histone modification coverage tracks were normalized to unmodified histone H4, and the RNAPII track was normalized to the corresponding input sample. All coverage tracks were visualized using IGV (70). Metaplots and heat maps were made using computeMatrix, plotProfile, and plotHeat map tools as part of the deepTools2 package (69).

10.1128/mBio.01376-19.7 FIG S7 Heat maps of pairwise correlations between different ChIP-seq and RNA-seq samples. The Pearson correlation coefficients are shown. The gradient red-to-blue color indicates the strength of correlation between the corresponding samples. (A) H3K9Ac, H4K16Ac, H2AS129P, H3K4me^3^, H3 and H4 IPs in WT strains; (B) H3K9Ac, H4K16Ac IP in WT and *sir2 Δ/Δ* strains; (C) H3K4me^3^ IP in WT and *set1 Δ/Δ* strains; (D) RNA-seq WT and *sir2 Δ/Δ* strains; (E) RNA-seq in WT and *set1 Δ/Δ* strains. Download FIG S7, TIF file, 1.7 MB. Copyright © 2019 Price et al. 2019 Price et al. This content is distributed under the terms of the Creative Commons Attribution 4.0 International license.

**(ii) q-ChIP-seq analysis.** To isolate the reads that uniquely aligned to the C. albicans genome, the full data sets were first aligned to the S. cerevisiae genome (sacCer3). The unaligned reads were outputted as separate fastq files, and these reads were then aligned to a custom haploid version of assembly 22 of the C. albicans genome (Table S2). The same strategy was used to isolate reads that uniquely aligned to S. cerevisiae (Table S2). All alignments were performed using Bowtie2 (66). The unique S. cerevisiae reads were then used to calculate the normalization factor (normalization factor = 1/[number of unique reference reads/1,000,000]), according to the method described previously by Orlando et al. (48). Reads that mapped to repeated sequences in the C. albicans genome were randomly assigned to copies of that repeat. Peak calling was performed using MACS2 (67) and the default settings, except that no model was used with all reads extended to 250 bp. MACS2 was run separately on the two biological replicates for each ChIP-seq sample. For each sample analyzed with MACS2, the IP sample was the “treatment,” and the input sample was the “control.” Peaks called in both replicate data sets for mutant and WT samples were combined into one peak set for each histone modification. Read counts within these peak intervals were generated using featureCounts (68), which were then scaled by the normalization factor to obtain the reference reads per million (RRPM). For each interval, RRPM values were compared between the mutant and WT samples using a two-sample *t* test, with a *P* value threshold of <0.05 being used to identify significant differences. Replicates were compared by generating a raw alignment coverage track and performing a Pearson correlation between them using the multiBamSummary and plotCorrelation tools as part of the deepTools2 package (Fig. S7) (69). Genome coverage tracks were made using the pileup function of MACS2 (67), and RRPM values were calculated for each track using the normalization factor. Data corresponding to coverage tracks from biological replicates were averaged after the replicates were deemed to be sufficiently correlative (*r* > 0.9), and the mutant strain track coverage data were normalized to the WT coverage. All tracks were visualized using IGV (70). Metaplots and heat maps were made using computeMatrix, plotProfile, and plotHeat map tools as part of the deepTools2 package (69).

**(iii) RNA-seq analysis.** Reads were aligned to a custom haploid version of assembly 22 of the C. albicans genome using HISAT2 (Table S2) (71), and per-gene transcript quantification was performed using featureCounts, which discards multimapped read fragments; therefore, only uniquely mapped reads were included for the expression analysis (68). Differential expression testing was performed using DESeq2, with an adjusted *P* value threshold of <0.05 being used to determine statistical significance. Replicates were compared by generating a raw alignment coverage track and performing a Pearson correlation between them using the multiBamSummary and plotCorrelation tools as part of the deepTools2 package (see Fig. S7) (69). Scatterplots and correlation analyses were performed in R using Pearson correlation.

### Data accessibility.

Raw data sets generated and analyzed during this study are available in the BioProject NCBI repository (https://www.ncbi.nlm.nih.gov/bioproject) under BioProject identifier (ID) PRJNA503946. Coverage tracks and genome files used in our analyses are available at https://www.kentfungalgroup.com/price-2019-data.
