# Supplementary figures and images for: Chromatin Profiling of the Repetitive and Nonrepetitive Genomes of the Human Fungal Pathogen Candida albicans
Source: mBio. 2019 Jul 23;10(4):e01376-19. doi: 10.1128/mBio.01376-19 (PMC6650553; doi:10.1128/mBio.01376-19)

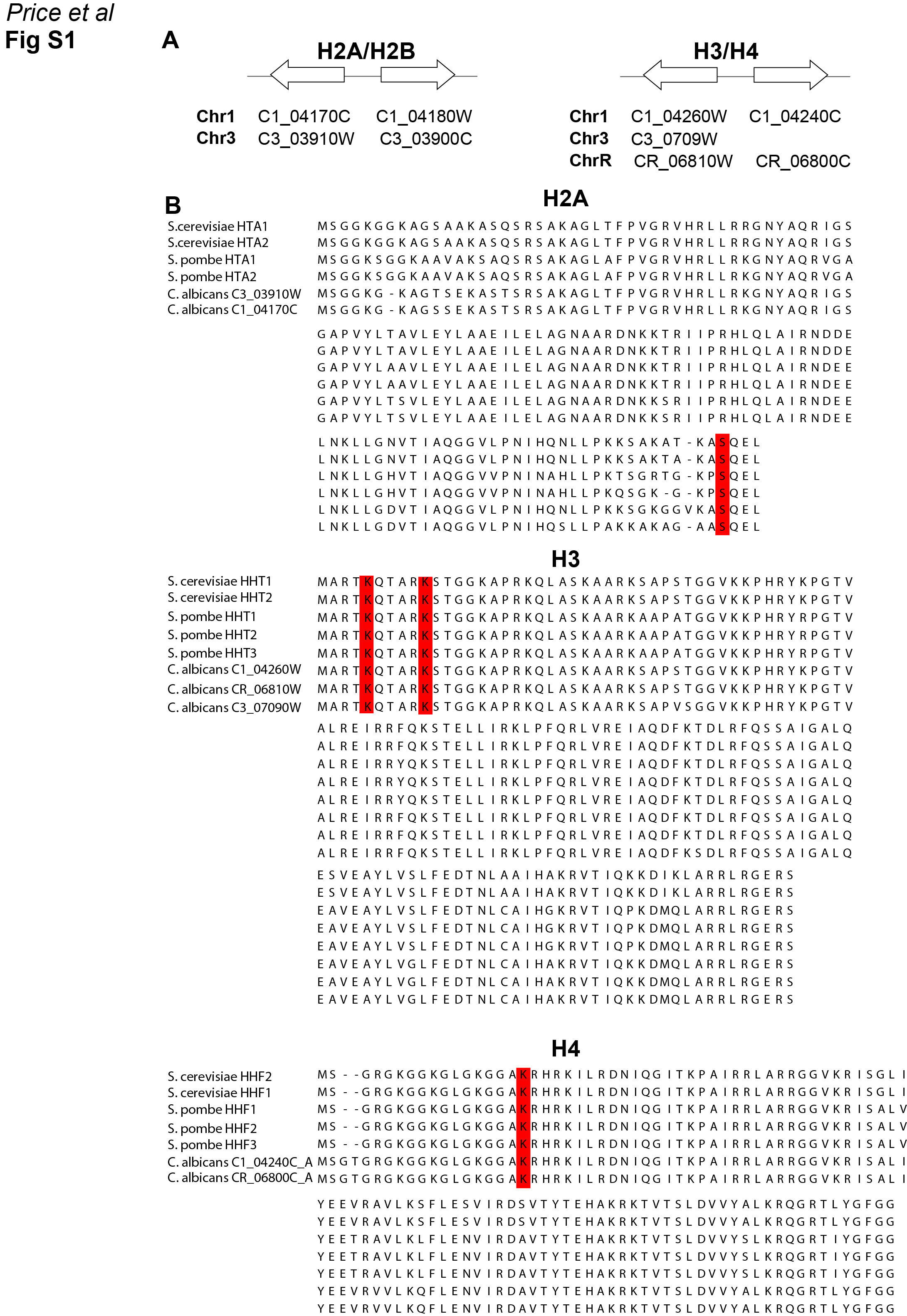

Supplement: FIG S1 [file mBio.01376-19-sf001.tif]

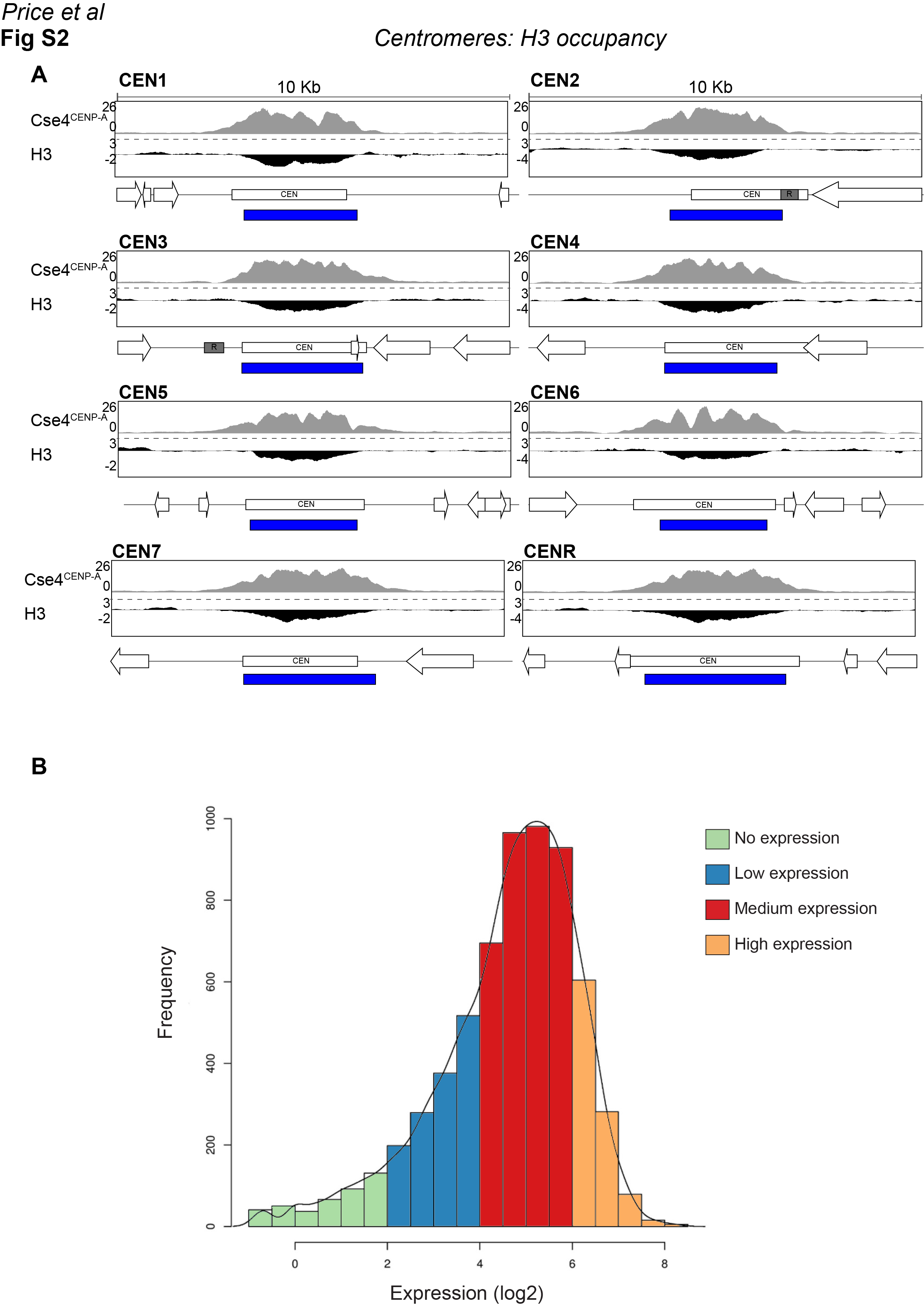

Supplement: FIG S2 [file mBio.01376-19-sf002.tif]

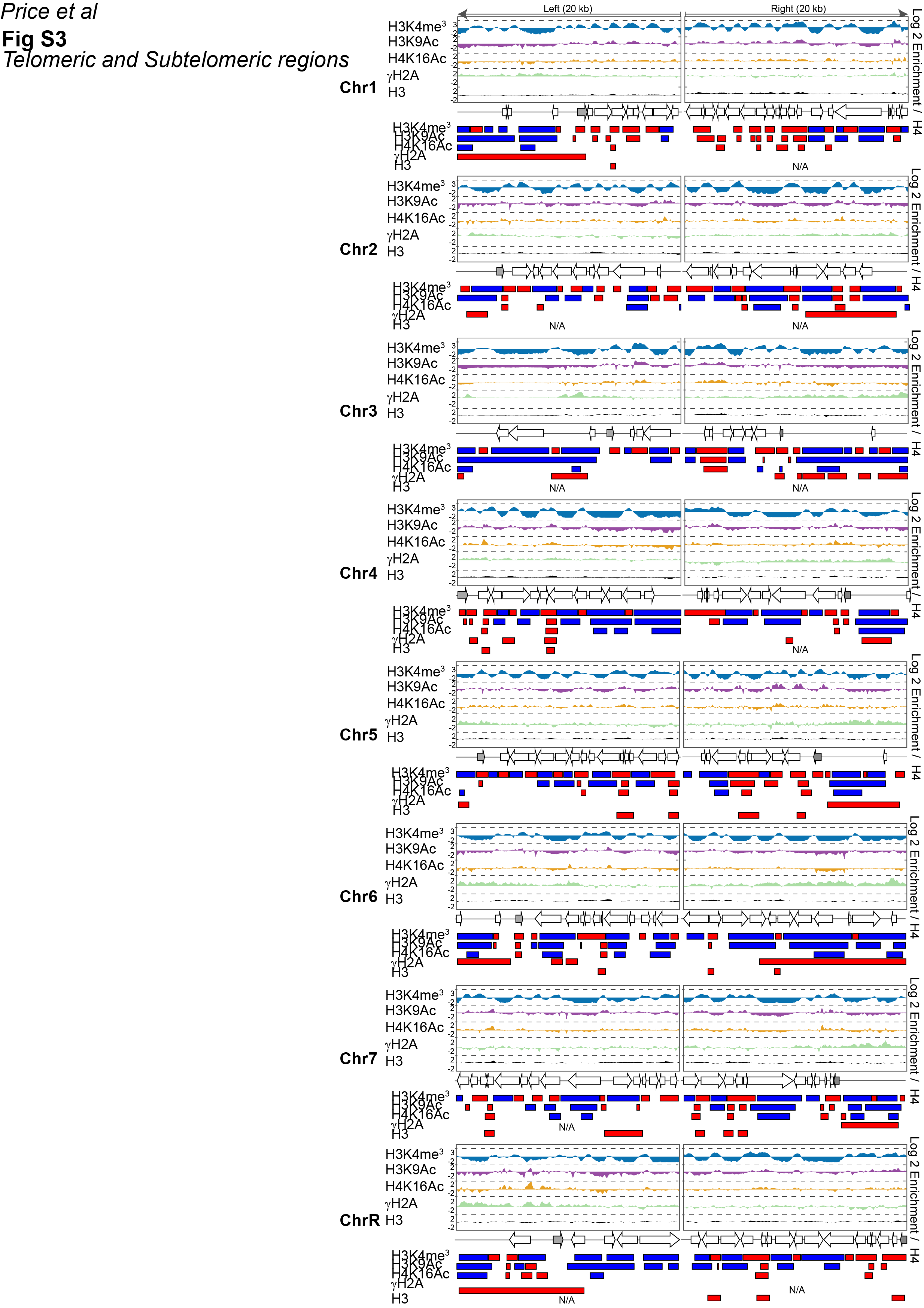

Supplement: FIG S3 [file mBio.01376-19-sf003.tif]

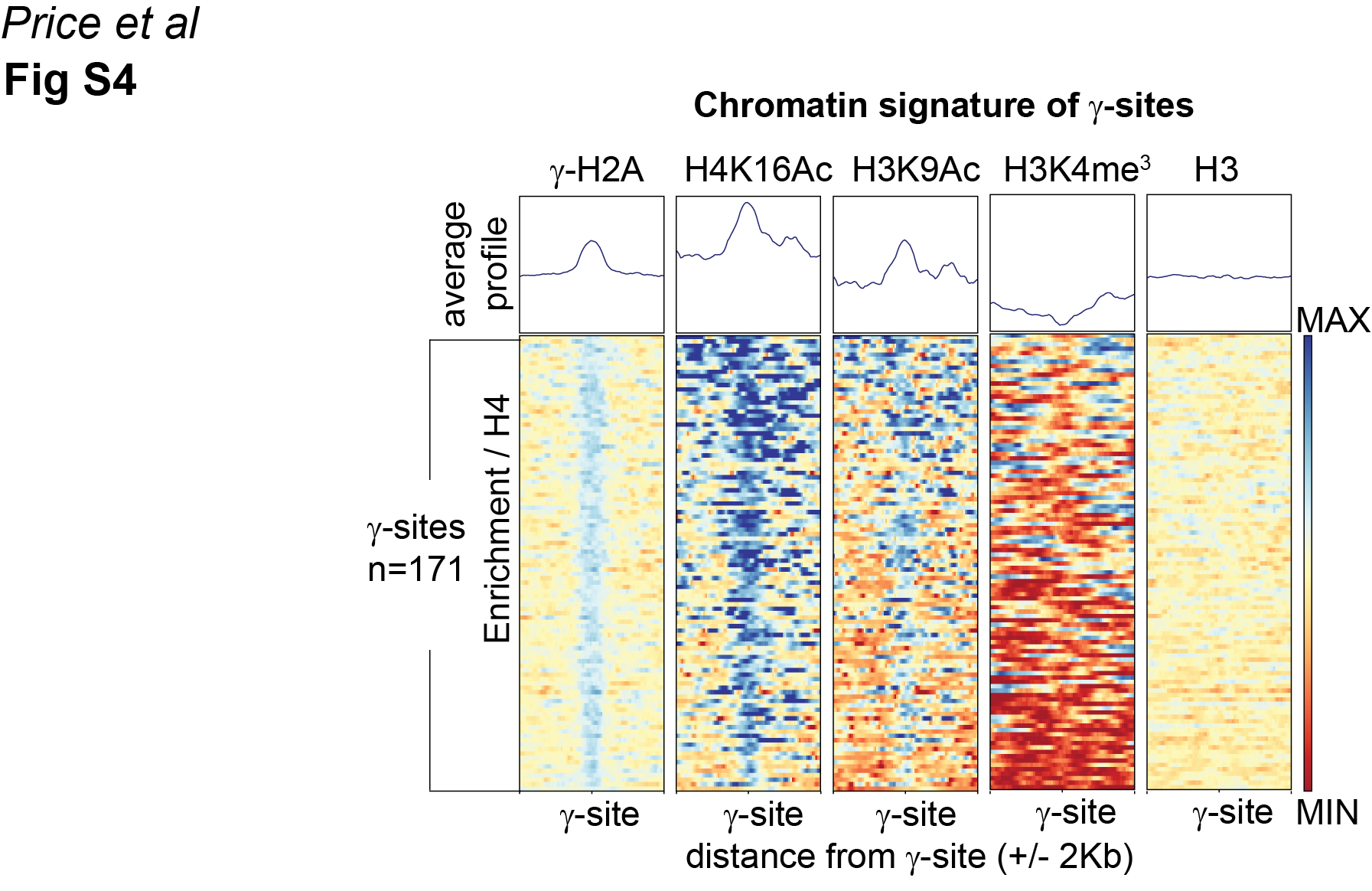

Supplement: FIG S4 [file mBio.01376-19-sf004.tif]

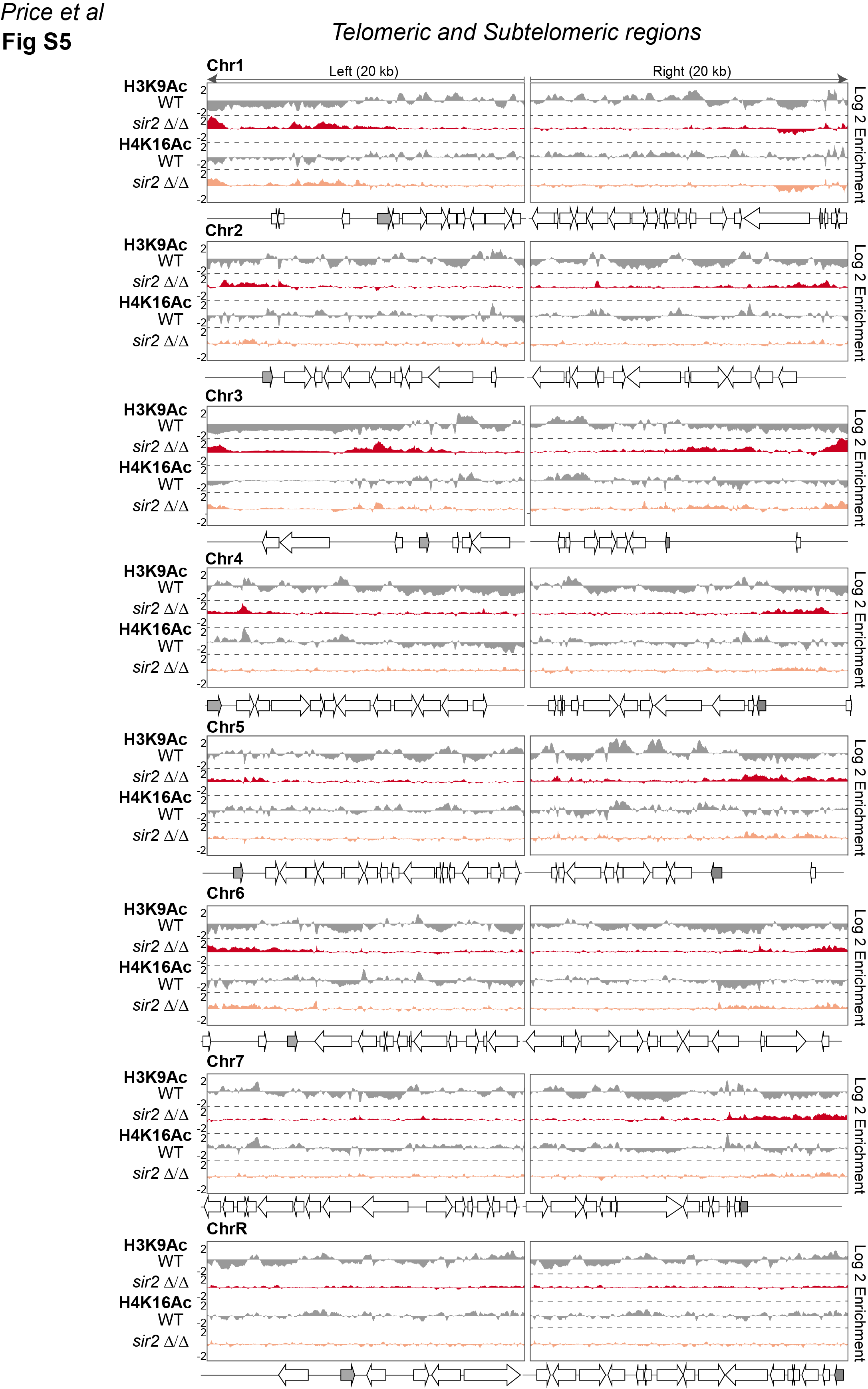

Supplement: FIG S5 [file mBio.01376-19-sf005.tif]

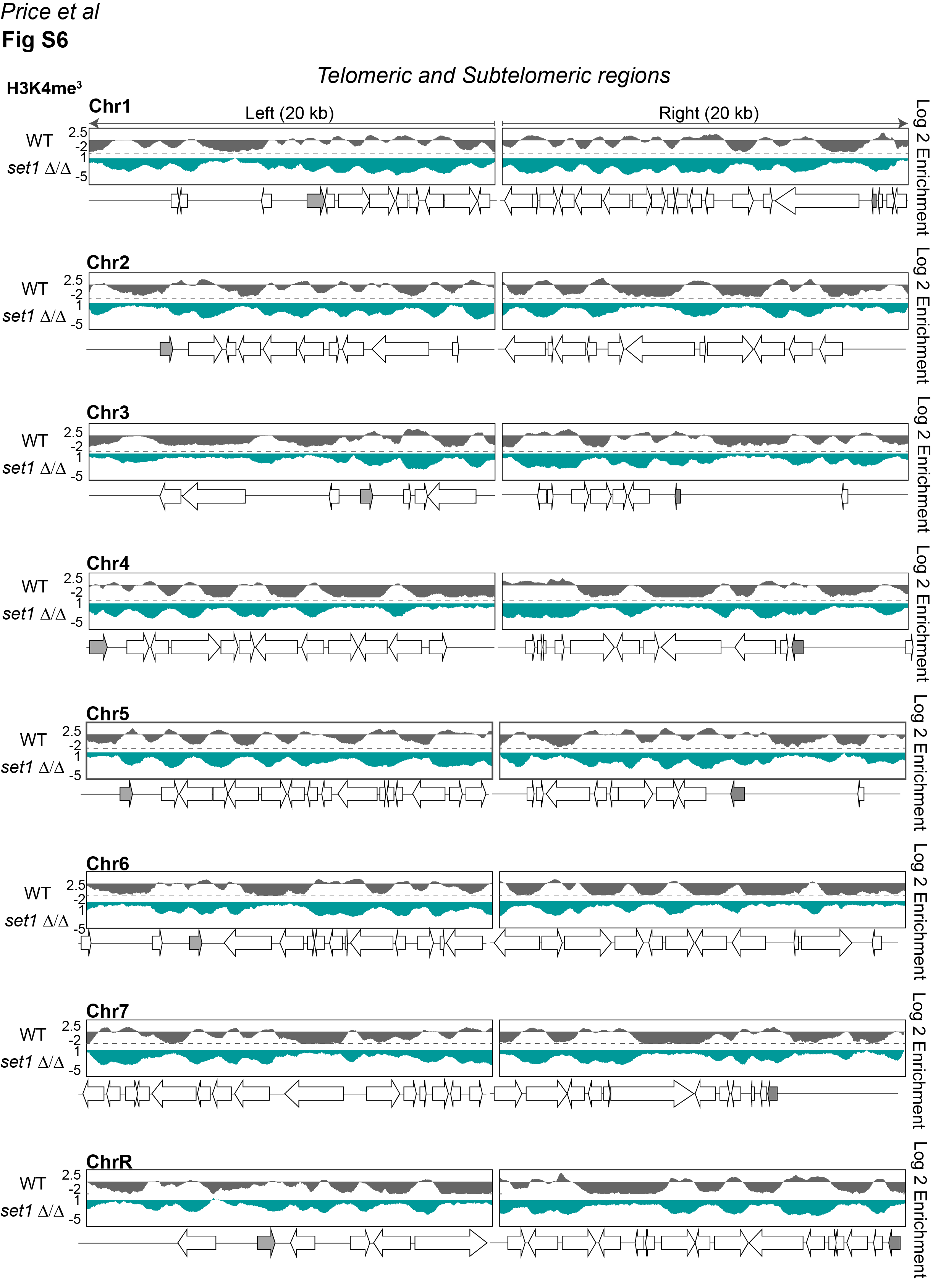

Supplement: FIG S6 [file mBio.01376-19-sf006.tif]

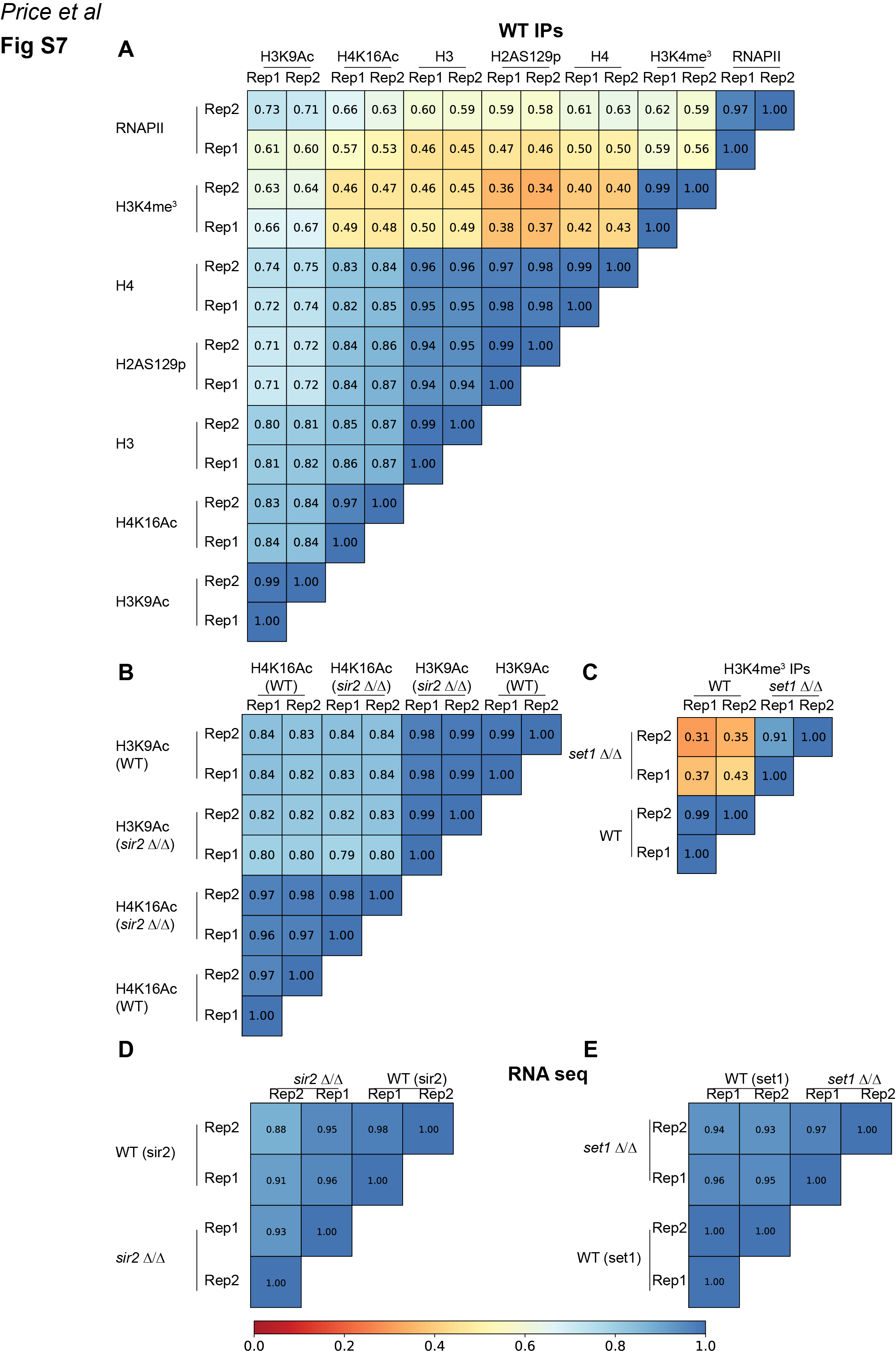

Supplement: FIG S7 [file mBio.01376-19-sf007.tif]
