# Supplementary material for: Chromatin Profiling of the Repetitive and Nonrepetitive Genomes of the Human Fungal Pathogen Candida albicans
Source: mBio. 2019 Jul 23;10(4):e01376-19. doi: 10.1128/mBio.01376-19 (PMC6650553; doi:10.1128/mBio.01376-19)
Supplement: TABLE S1 [file mBio.01376-19-st001.docx]

| **Name** | **Species** | **Strain** | **Genotype** |
| --- | --- | --- | --- |
| WT (BWP17) | *C. albicans* | AB215 | ura3Δ::Δimm434/ura3Δ::Δimm434 his1::hisG/his1::hisG arg4::hisG/arg4::hisG |
| *sir2 Δ/Δ* | *C. albicans* | AB20 | ura3Δ::λimm434/ura3Δimm434 his1::hisG/his1::hisG arg4::hisG/arg4::hisG sir2::HIS1/sir2::ARG4 |
| *set1 Δ/Δ* | *C. albicans* | AB169 | ura3Δ::λimm434/ura3Δ::λimm434  his1::hisG/his1::hisG  arg4::hisG/arg4::hisG set1::HIS1/set1::ARG4 |
| WT | *S. cerevisiae* | BY4741 | MATa his3Δ1 leu2Δ0 met15Δ0 ura3Δ0 |
| URA3-rDNA | *C. albicans* | AB97 | ura3*Δ::λimm434/ura3Δimm434 his1::hisG/his1::hisG arg4::hisG/arg4::hisG URA3-rDNA* |
| rDNA-URA3 | *C. albicans* | AB185 | *ura3Δ::imm434 ura3Δ::imm434 his1::hisG/his1::hisG ERG13/ERG13-GFP-URA3* |
| Tel5 | *C. albicans* | AB148 | *ura3Δ::imm434/ura3Δ03::imm434 his1::hisg/his1::hisG arg4::hisG/arg4::hisG CTA24/CTA24-GFP-URA3-tADH* |
| Tel4 | *C. albicans* | AB147 | *ura3Δ::imm434 ura3Δ::imm434 his1::hisG/his1::hisG arg4::hisG/arg4::hisG TLOα10/ TLOα10-GFP-URA3-tADH* |
| Tel7 | *C. albicans* | AB253 | *ura3Δ::imm434 ura3Δ::imm434 his1::hisG/his1::hisG arg4::hisG/arg4::hisG TLO* γ*16/TLO* γ*16-GFP-URA3* |
| GAL1 | *C. albicans* | AB652 | *ura3*Δ::λimm434/*ura3*Δ::λimm434 *his1*::hisG/*his1*::*hisG gal1*::*URA3*/*GAL1*, *HIS1* |
| SAP5 | *C. albicans* | AB661 | *ura3*Δ::λimm434/*ura3*Δ::λimm434 *his1*::hisG/*his1*::*hisG sap5*::*URA3*/*SAP5* |
| HCM1 | *C. albicans* | AB659 | *ura3*Δ::λimm434/*ura3*Δ::λimm434 *his1*::hisG/*his1*::*hisG hcm1*::*URA3*/*HCM1* |
| CLB4 | *C. albicans* | AB655 | *ura3*Δ::λimm434/*ura3*Δ::λimm434 *his1*::hisG/*his1*::*hisG clb4*::*URA3*/*CLB4* |
| URA3 | *C. albicans* | AB54 | *MTL a/alpha ura3Δ-iro1Δ::imm434/URA3-IRO1 his1Δ/his1Δ arg4Δ/arg4Δ leu2Δ/leu2Δ* |
| PHO85 | *C. albicans* | AB653 | *ura3*Δ::λimm434/*ura3*Δ::λimm434 *his1*::hisG/*his1*::*hisG pho85*::*URA3*/*PHO85* |

**Table S1**: Strains used in this study
